# Sex-Related Differences in Skin Temperature Responses Induced by Small-Sided Games Assessed Using Infrared Thermography

**DOI:** 10.3390/jfmk11040397

**Published:** 2026-10-01

**Authors:** Marvyn Moya Ortega, Estefanía Estrada Ocampo, Juan David Fernández Villada, John Garcia Tamayo, Diego Gómez Bedoya, Angel Martínez del Águila, Walter Gómez-Sánchez

**Affiliations:** 1 Management, Administration and Sports Communication Research Group (GESTAS), Faculty of Physical Education, Recreation and Sports, Politécnico Colombiano Jaime Isaza Cadavid, Medellín 050022, Colombia; estefania_estrada80201@elpoli.edu.co (E.E.O.); jdfernandez@elpoli.edu.co (J.D.F.V.); jagarcia@elpoli.edu.co (J.G.T.); diego_gomez84103@elpoli.edu.co (D.G.B.); wlgomez@elpoli.edu.co (W.G.-S.); 2 Institute of Physical Education, University of Antioquia, Medellín 050010, Colombia; angelmaguila76@gmail.com

**Keywords:** infrared thermography, skin temperature, thermal asymmetry, soccer, sex differences, small-sided games

## Abstract

**Introduction**: Infrared thermography (IRT) enables non-invasive assessment of exercise-induced changes in skin temperature (Tsk); however, evidence on sex-related responses to soccer-specific exercise remains limited. This study compared Tsk responses and bilateral thermal asymmetry between women and men soccer players following a small-sided game (SSG). **Methods**: This repeated-measures experimental study included 46 university soccer players (22 women, 24 men), who completed a standardised 4 vs. 4 SSG. Thermographic images were obtained before (Pre), approximately 5 min after exercise (Post), and 10 min after the Post assessment (Post_10). Tsk was assessed in eight lower-limb regions of interest (ROIs). Linear mixed-effects models examined Sex × Time effects, with participant included as a random intercept. **Results**: Significant Sex × Time interactions were observed across all eight ROIs (Holm-adjusted *p* < 0.001). Women showed greater increases in Tsk than men from Pre to Post (between-sex differences: 0.86–1.55 °C) and greater net changes from Pre to Post_10 (1.16–1.70 °C). No between-sex differences were observed for the change from Post to Post_10 (all Holm-adjusted *p* > 0.05). Bilateral thermal asymmetry showed no significant Sex × Time interactions (all *p* ≥ 0.390). **Conclusions**: Women and men exhibited different Tsk responses following the SSG, particularly at the Post assessment and in the net change from Pre to Post_10, whereas changes during the 10 min recovery interval were comparable between sexes. Bilateral thermal asymmetry also showed comparable temporal patterns. These findings highlight the importance of considering sex and assessment timing when interpreting exercise-induced Tsk responses using IRT.

## 1. Introduction

Soccer is an intermittent sport characterised by repeated high-intensity actions, including accelerations, decelerations, changes in direction, jumps, and sprints, which impose substantial physiological and neuromuscular demands during training and competition [1,2]. During exercise, a considerable proportion of the metabolic energy produced is released as heat, requiring continuous adjustments in heat production and dissipation to maintain thermal balance [3,4]. Consequently, skin temperature (Tsk) reflects the interaction of several physiological processes occurring at the body surface and may change substantially during and after exercise.

In recent years, infrared thermography (IRT) has become established as a non-invasive and rapid technique for assessing the spatial distribution of Tsk in athletes [5]. Its growing application in sport science has enabled the assessment of regional Tsk changes associated with exercise, training load, and post-exercise responses [6,7]. Recent evidence from endurance exercise further supports the ability of IRT to characterise acute and region-specific Tsk responses. In competitive race walking, post-exercise Tsk decreased across several lower-limb regions while increasing in the posterior leg, highlighting the heterogeneous regional response to exercise [8]. Moreover, during ramp-incremental cycling exercise, changes in calf Tsk were statistically associated with intensity-dependent changes in metabolism, with the thermal breakpoint showing closer correspondence with the gas exchange threshold than with the respiratory compensation point [9]. More recently, a systematic review highlighted the potential of IRT for assessing skin temperature, fatigue-related responses, thermal asymmetry, and recovery in soccer players [10]. However, the interpretation of exercise-induced Tsk responses is complex because thermographic measurements may be influenced by multiple physiological, individual, environmental, and methodological factors.

Within soccer, small-sided games (SSGs) are widely used because they simultaneously reproduce several physical, physiological, technical, and tactical demands of match play [11,12]. These tasks provide an ecologically relevant context in which to examine exercise-induced thermal responses. Previous research has shown that IRT can detect changes in the thermal profile of soccer players following SSGs [13]. Nevertheless, a recent systematic review highlighted the limited evidence regarding the influence of biological variables, including sex, on thermal responses in soccer players [10].

Sex represents an important biological factor that may contribute to variability in exercise-induced Tsk responses. Differences in body composition, muscle mass, adipose tissue percentage, body surface area-to-mass ratio, and cutaneous blood flow regulation may affect heat production and heat dissipation during exercise [4,14]. Although previous studies have reported sex-related differences in resting skin temperature and post-exercise thermal responses [15,16], evidence remains scarce in soccer-specific settings. In particular, relatively few studies have directly compared women and men exposed to the same soccer-specific exercise protocol. Consequently, it remains unclear whether women and men soccer players exhibit different magnitudes and temporal patterns of Tsk response following SSGs.

An additional unresolved issue concerns the temporal evolution of these responses after exercise. Potential sex-related differences may be evident during the early post-exercise period and may change during subsequent recovery. Therefore, assessing Tsk at different time points during the early post-exercise period may provide a more complete description of the temporal thermal response. Furthermore, whether bilateral thermal asymmetry exhibits different temporal patterns between women and men following soccer-specific exercise remains unclear.

Importantly, exercise-induced Tsk responses should not be interpreted independently of the physical and physiological demands of exercise. Differences in internal and external load, body composition, and other individual factors may contribute to the magnitude and temporal pattern of Tsk responses. In women, the menstrual cycle phase may represent an additional source of variability. Therefore, sex-related differences in Tsk following soccer-specific exercise should be interpreted within this multifactorial context.

Therefore, the aim of the present study was to compare changes in skin temperature and bilateral thermal asymmetry between women and men university soccer players before, approximately 5 min after, and 10 min after the Post assessment following a standardised SSG using IRT. We hypothesised that women players would exhibit greater increases in skin temperature during both the early post-exercise response and the subsequent recovery period following the SSG, whereas thermal asymmetry would remain similar between sexes.

## 2. Materials and Methods

### 2.1. Study Design

A repeated-measures experimental design was used, with Sex (women and men) as the between-subject factor and Time as the within-subject factor. Skin temperature was assessed before the SSG (Pre), approximately 5 min after completion of the SSG (Post), and 10 min after the Post assessment (Post_10). The dependent variables were changes in skin temperature (ΔTsk) and bilateral thermal asymmetry calculated for the different regions of interest (ROIs) of the lower limbs.

### 2.2. Participants

Forty-seven university soccer players were initially enrolled in the study. One woman did not complete the study protocol and was therefore excluded from the final analysis. The final analytical sample comprised 46 university soccer players (22 women and 24 men) from university representative teams. Body fat percentage was assessed at baseline using bioelectrical impedance analysis (BIA) with a Tanita BC-418 MA body composition analyzer (Tanita Corp., Tokyo, Japan) (Table 1). The group-level mean and standard deviation reported in Table 1 were calculated from the individual measurements during the original data-processing stage. Because body fat percentage was used solely for descriptive characterisation of the sample and was not included in the inferential analyses, the individual body fat percentage values were subsequently removed from the analytical database and were not retained for additional analyses. All players had at least five years of competitive soccer experience, trained at least three times per week, and regularly competed in official matches.

The inclusion criteria were: (i) regular participation in training sessions and competitive matches; (ii) absence of musculoskeletal injuries during the three months preceding data collection; and (iii) completion of all experimental assessments. Participants were excluded if they presented with an acute illness, sustained an injury during the experimental protocol, or failed to comply with the pre-assessment thermographic recommendations.

The sample size was determined by convenience based on the availability of eligible university soccer players who met the inclusion criteria and completed the experimental protocol. No a priori sample size calculation was performed.

Before data collection, all participants received detailed information regarding the study procedures and provided written informed consent. The study protocol was conducted in accordance with the Declaration of Helsinki and was approved by the Ethics Committee of the Politecnico Colombiano Jaime Isaza Cadavid (under Min No. 20, dated 6 December 2024).

### 2.3. Procedures

All participants attended a single experimental session conducted on the institution’s soccer field between 10:00 and 12:00 h to minimise the influence of circadian variation on skin temperature.

Before testing, participants were instructed to refrain from vigorous physical exercise for 24 h, avoid caffeine, alcohol, and other stimulants for 12 h, and refrain from applying creams, lotions, or cosmetic products to the skin on the day of testing. They were also instructed to maintain their usual dietary and hydration habits.

Upon arrival, participants underwent a 15 min acclimation period in the controlled assessment environment to allow thermal stabilisation. During the final 10 min of this period, participants remained seated at rest before the baseline thermographic images (Pre) were acquired.

Following thermal stabilisation, baseline thermographic images (Pre) were acquired using a standardised body positioning and image acquisition protocol. Participants then completed a standardised warm-up before performing the SSG. After completion of the final SSG bout, participants immediately moved from the playing field to the laboratory, which required approximately 3 min. Approximately 2 additional minutes were required for the preparation and initiation of thermographic image acquisition; thus, the Post assessment began approximately 5 min after completion of the SSG. Following 10 min of passive recovery after the Post assessment, a third thermographic assessment (Post_10) was obtained.

All thermographic images acquired at the three assessment time points were analysed using the same processing procedure to determine the mean skin temperature of each ROI. These values were subsequently used to calculate changes in skin temperature between time points (ΔPost–Pre, ΔPost_10–Post, and ΔPost_10–Pre), as well as bilateral thermal asymmetry between the right and left sides of the body.

**Figure 1:**
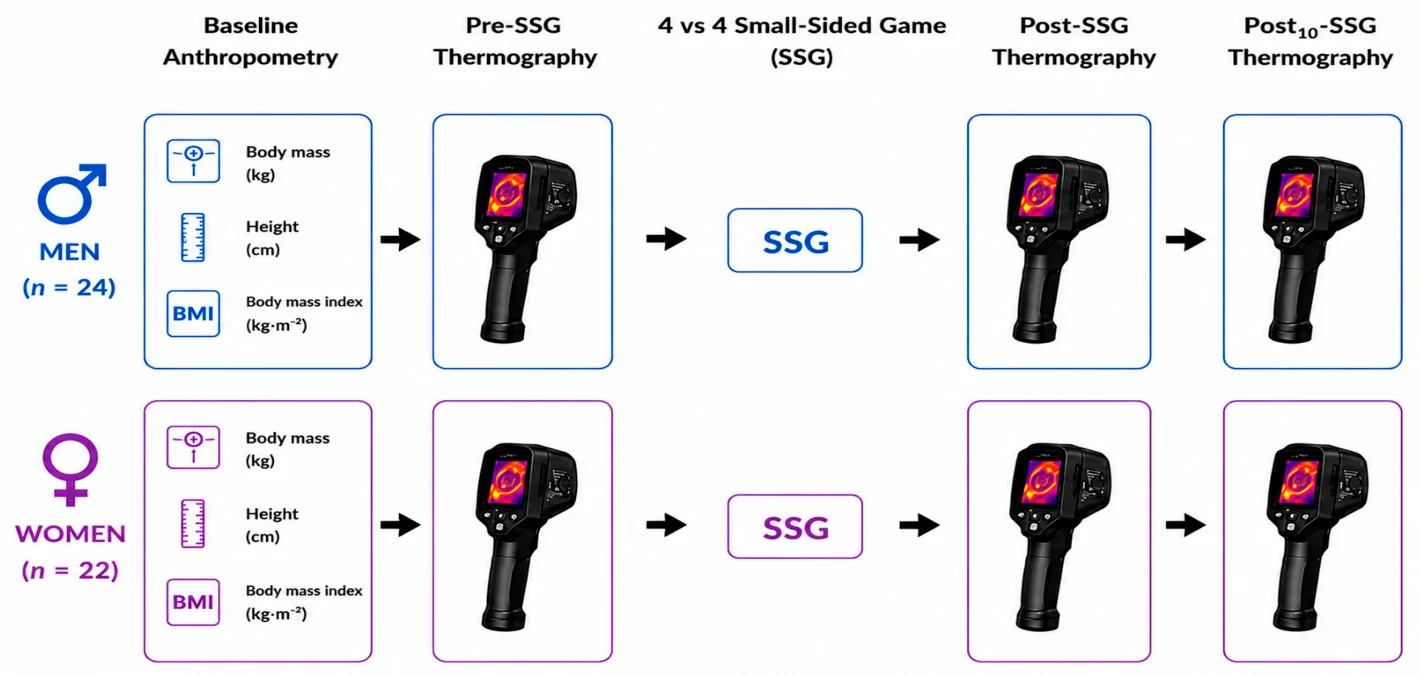
Schematic representation of the experimental protocol.

The experimental protocol was conducted in the same assessment environment and supervised by the same investigators to promote consistency and standardisation throughout all measurements (Figure 1).

### 2.4. Assessment of Skin Temperature

Following the 15 min acclimation period described above, during which participants remained seated at rest for the final 10 min, thermographic images were acquired using an infrared camera (HIKMICRO M31T, 384 × 288 pixels; thermal sensitivity [NETD] < 0.05 °C; accuracy ± 0.5 °C; HIKMICRO, Hangzhou, China) and analysed using HIKMICRO Analyzer software (HIKMICRO, Hangzhou, China). The camera was switched on 10 min before image acquisition to ensure sensor stabilisation and positioned 1 m from the participant, perpendicular to the ROI.

During the SSG, all participants wore shorts, a sleeveless shirt, and short socks. After the final SSG bout, the sleeveless shirt and socks were removed before the Post thermographic assessment. During thermographic image acquisition, participants were barefoot and standing in the anatomical position; men remained wearing shorts, whereas women remained wearing shorts and a sports top. No additional 10–15 min post-exercise thermal stabilisation period was implemented before the Post assessment [17]. For all measurements, ambient temperature and relative humidity were monitored using a digital thermo-hygrometer (TFA Dostmann, Wertheim-Reicholzheim, Germany), positioned in the laboratory adjacent to the evaluator. The recorded environmental conditions were approximately 26 °C and 86% relative humidity, and these values were entered into the infrared camera settings before image acquisition.

Furthermore, participants followed the pre-assessment recommendations for thermographic imaging, including abstaining from alcohol, caffeine, and tobacco consumption, vigorous physical exercise, prolonged sun exposure, large meals, and the application of creams, ointments, or cosmetic products before testing to minimise factors that could influence skin temperature [18]. Compliance with these recommendations was verified using the Thermographic Imaging in Sports and Exercise Medicine (TISEM) checklist [18].

**Figure 2:**
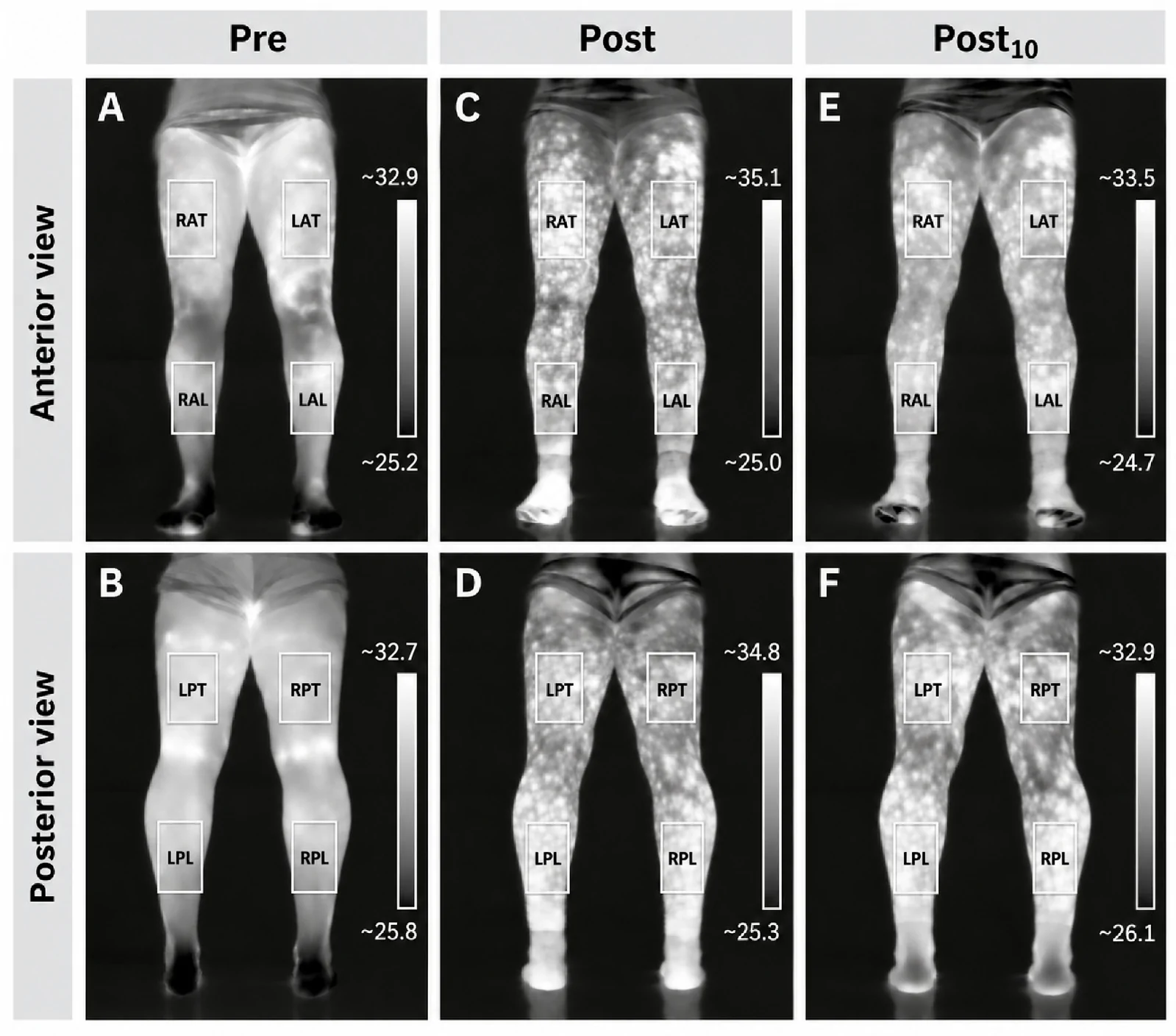
Representative infrared thermographic images of the anterior and posterior lower limbs from the same participant obtained before the small-sided game (Pre) (**A**,**B**), approximately 5 min after the small-sided game (Post) (**C**,**D**), and 10 min after the Post assessment (Post_10) (**E**,**F**). Images were acquired according to the standardized thermographic protocol described in the Methods. Regions of interest (ROIs) used for skin temperature analysis. The ROIs included the right anterior thigh (RAT), left anterior thigh (LAT), right anterior leg (RAL), left anterior leg (LAL), right posterior thigh (RPT), left posterior thigh (LPT), right posterior leg (RPL), and left posterior leg (LPL).

Four ROIs of the lower limbs were analysed: anterior thigh (AT), posterior thigh (PT), anterior leg (AL), and posterior leg (PL) (Figure 2). Mean skin temperature (°C) was recorded for each ROI at the three experimental time points (Pre, Post, and Post_10). These measurements were subsequently used to calculate skin temperature changes and bilateral thermal asymmetries.

### 2.5. Calculation of Skin Temperature Changes and Thermal Asymmetry

Skin temperature changes were calculated from the mean skin temperature recorded in each ROI at the different assessment time points, as follows:

- ΔTsk (Post–Pre) = Post skin temperature − Pre skin temperature.
- ΔTsk (Post_10–Post) = Post_10 skin temperature − Post skin temperature.
- ΔTsk (Post_10–Pre) = Post_10 skin temperature − Pre skin temperature.

Bilateral thermal asymmetry was determined as the absolute difference in skin temperature between the right and left homologous anatomical regions using the following equation:

Thermal asymmetry = |TRight − TLeft|

where T Right and T Left: represent the mean skin temperature of the right and left homologous ROIs, respectively.

### 2.6. Small-Sided Game

The intervention consisted of a 4 vs. 4 SSG performed on a 30 × 15 m pitch, providing an individual playing area of 56 m^2^ per player, with small goals. The offside rule was not applied, and ball replacement was performed manually whenever the ball left the playing area [19]. To minimise interruptions and maintain continuous play, more than four balls were strategically positioned around the pitch to allow rapid ball replacement. The protocol consisted of five 3 min bouts, interspersed with 1 min passive recovery periods.

No verbal encouragement was provided by the investigators during the SSG. Teams were selected by the head coach to ensure a balanced distribution of technical ability, tactical competence, and playing positions. Each team included at least one defender, one midfielder, and one forward. Team compositions were predetermined and remained unchanged throughout all experimental sessions to optimise team dynamics and ensure consistency between assessments.

Exercise intensity during the intervention was continuously monitored using Sigma Sport iD.RUN HR heart rate monitors (Sigma Sport, Germany). Age-predicted maximal heart rate (HRmax) was estimated using the equation HRmax = 208 − (0.7 × age) [20]. Participants were required to reach at least 95% of their age-predicted HRmax by the end of each 3 min work bout. This criterion was verified in real time by the researchers from the heart-rate values displayed by the monitoring system during the final part of each bout. Heart rate was used exclusively as a real-time control of exercise intensity and was not treated as an outcome variable; individual heart-rate recordings were not exported or retained for subsequent analysis. All participants included in the final analysis met the predefined intensity criterion during the monitored bouts. This threshold was used to verify that a high exercise intensity was achieved during the SSG and was not used as an exclusion criterion.

Immediately after completing the SSG, participants reported their session rating of perceived exertion (sRPE) using the Category Ratio-10 (CR-10) Borg scale by answering the question, “How intense was the session?” [21]. The CR-10 scale was administered individually to each participant to minimise the influence of peer responses. Although sRPE was recorded following the SSG, it was not included as an outcome in the present analysis.

### 2.7. Statistical Analysis

Statistical analyses were performed using Python (version 3.13.5), with statsmodels (version 0.14.6) used for the linear mixed-effects models and SciPy (version 1.17.0) for independent-samples Student’s *t*-tests. Continuous variables are presented as mean ± standard deviation (SD), and statistical significance was set at *p* < 0.05. Anthropometric characteristics were compared between women and men using independent-samples Student’s *t*-tests.

Skin temperature was analysed using separate linear mixed-effects models for each region of interest (ROI), with sex (women vs. men), time (Pre, Post, and Post_10), and their interaction (Sex × Time) included as fixed effects and participant as a random intercept. Models were fitted using restricted maximum likelihood (REML), and fixed effects were evaluated using Wald χ^2^ tests. Prespecified contrasts examined Post–Pre (change from baseline to the non-stabilised Post assessment obtained approximately 5 min after exercise), Post_10–Post (change during the 10 min recovery period), and Post_10–Pre (net change from baseline to Post_10). *p* values for multiple comparisons were adjusted using the Holm procedure.

Bilateral thermal asymmetry was analysed using the same mixed-model approach for the anterior thigh, posterior thigh, anterior leg, and posterior leg, with sex, time, and the Sex × Time interaction as fixed effects and participant as a random intercept.

## 3. Results

### 3.1. Changes in Skin Temperature

Significant Sex × Time interactions were observed across all eight ROIs (Wald χ^2^ = 18.26–34.16, all Holm-adjusted *p* < 0.001; Table 2). Post hoc contrasts showed greater Pre-to-Post increases in women than in men across all ROIs, with between-sex differences ranging from 0.86 to 1.55 °C (Holm-adjusted *p* ≤ 0.005). Similarly, women exhibited greater net changes from Pre to Post_10 (between-sex differences: 1.16–1.70 °C; all Holm-adjusted *p* < 0.001). In contrast, no significant between-sex differences were observed for the change occurring specifically during the Post-to-Post_10 recovery interval (between-sex differences: 0.13–0.48 °C; all Holm-adjusted *p* > 0.05).

### 3.2. Thermal Asymmetry

**Figure 3:**
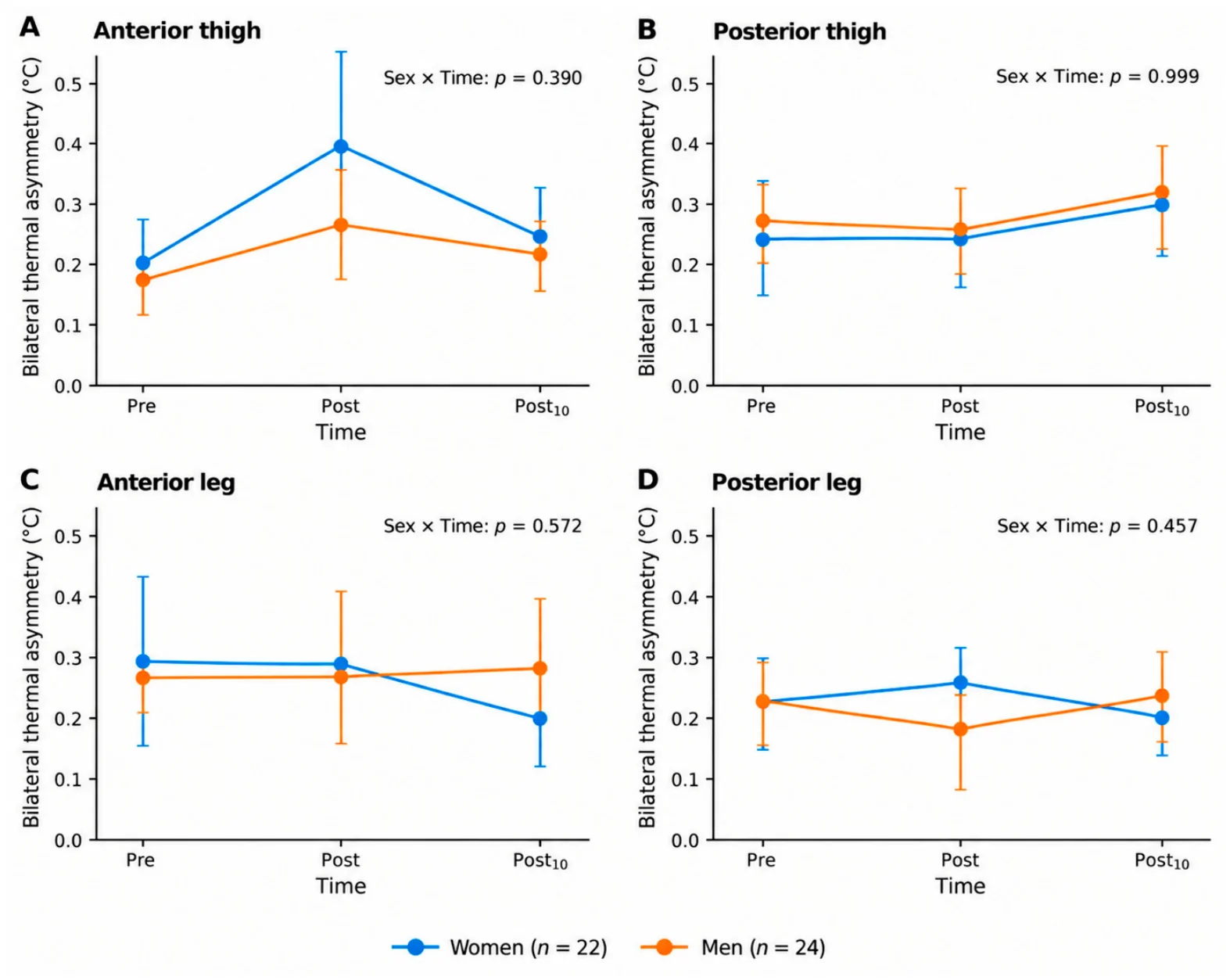
Temporal changes in bilateral thermal asymmetry in women and men following the small-sided game. Note. Values are presented as mean ± 95% confidence interval. Panels represent (**A**) anterior thigh, (**B**) posterior thigh, (**C**) anterior leg, and (**D**) posterior leg. Bilateral thermal asymmetry was calculated as the absolute difference in skin temperature between homologous right and left regions. *p* values correspond to the Sex × Time interaction from linear mixed-effects models with participant included as a random intercept. No significant Sex × Time interactions were observed in any anatomical region (*p* > 0.05).

No significant Sex × Time interactions were observed for bilateral thermal asymmetry in the anterior thigh (*p* = 0.390), posterior thigh (*p* = 0.999), anterior leg (*p* = 0.572), or posterior leg (*p* = 0.457; Figure 3). Overall, these findings indicate that bilateral thermal asymmetry showed comparable temporal patterns between women and men across the three assessment time points.

## 4. Discussion

The present study compared skin temperature responses and bilateral thermal asymmetry between women and men soccer players following a standardised SSG using IRT. The main findings were that significant Sex × Time interactions were observed across all eight ROIs, with women showing greater increases in skin temperature than men from Pre to the non-stabilised Post assessment obtained approximately 5 min after exercise, and greater net changes from baseline to Post_10 (Post_10–Pre). However, no significant between-sex differences were observed for the change occurring specifically during the 10 min recovery interval (Post_10–Post). Therefore, the hypothesis that women would exhibit greater skin temperature increases during both the approximately 5 min post-exercise assessment and subsequent recovery was only partially supported. In contrast, bilateral thermal asymmetry showed no significant Sex × Time interactions, indicating comparable temporal patterns between women and men.

These findings extend the current evidence regarding the application of IRT in soccer. Although IRT has been used to monitor exercise-induced skin temperature responses and post-exercise changes, most previous studies have been conducted in men soccer players or have not considered sex as an analytical variable [10,22]. Consequently, evidence regarding sex-related skin temperature responses remain limited, particularly in women players performing intermittent high-intensity tasks such as SSGs.

The greater increases in Tsk observed in women from Pre to the non-stabilised Post assessment obtained approximately 5 min after exercise, as well as the greater net changes from Pre to Post_10, should not be attributed exclusively to sex-specific thermoregulatory or vascular mechanisms. Skin temperature does not directly reflect muscle heat production but rather represents the net result of the dynamic interplay between metabolic heat production, cutaneous blood perfusion, and heat dissipation mechanisms [10,22]. Although heart rate was continuously monitored in real time to control exercise intensity, individual heart-rate recordings were not retained or analysed as an internal-load outcome, and external-load variables such as total distance, high-speed running, accelerations, and decelerations were not quantified. Furthermore, body fat percentage differed between sexes, and menstrual cycle phase was not controlled.

These factors may have influenced the observed Tsk responses and should therefore be considered potential confounders. Because core temperature, cutaneous blood flow, sweating responses, and metabolic heat production were not directly measured, the underlying physiological mechanisms cannot be established. Consequently, the present findings should be interpreted as observed differences in the temporal pattern of skin temperature between women and men under the experimental conditions of this study, rather than as evidence of an independent effect of biological sex on thermoregulatory function.

Our findings partially agree with those reported by [23], who observed that although men players exhibited higher absolute skin temperatures following the FIFA 11+ protocol, women players showed greater relative thermal changes. Similarly, in the present study, women exhibited greater exercise-induced changes in skin temperature than men across several lower-limb regions. These findings suggest that sex may influence the magnitude and temporal pattern of skin temperature responses following intermittent exercise. However, the physiological mechanisms underlying these differences remain incompletely understood.

Several physiological factors may contribute to the observed sex-related differences in skin temperature responses. Differences in body composition, body surface area-to-mass ratio, cutaneous blood flow regulation, and sudomotor function may influence the transfer and dissipation of exercise-generated heat at the skin surface [24]. However, these mechanisms were not directly assessed in the present study. Skin temperature responses may also be influenced by relative exercise intensity, training status, and environmental conditions. Therefore, sex should be interpreted as a factor associated with differences in the observed skin temperature response rather than as an independent causal mechanism.

A relevant finding was that between-sex differences remained evident when skin temperature at Post_10 was considered relative to baseline (Post_10–Pre). Importantly, this contrast represents the net change from baseline to Post_10 and should not be interpreted as the temperature change occurring specifically during recovery. Indeed, no significant between-sex differences were observed for the Post_10–Post contrast across any ROI. Although previous studies have suggested that skin temperature continues to evolve following exercise and that post-exercise thermal kinetics may provide relevant physiological information [22,25], the present findings do not demonstrate greater physiological sensitivity at Post_10. Rather, they indicate that the sex-related differences observed in the overall temperature response remained evident at Post_10. Further studies incorporating additional post-exercise measurements are required to determine whether the recovery kinetics of skin temperature differ between women and men soccer players.

Furthermore, sex-related differences in skin temperature responses were observed across both anterior and posterior regions of the thigh and lower leg, indicating that these differences were not restricted to a single anatomical region. Previous studies have shown that regional skin temperature responses may vary according to the type of exercise performed and the anatomical region assessed [23,26]. This regional variability is also consistent with recent findings in competitive race walking, where Tsk decreased across several lower-limb regions after competition but increased in the posterior leg, further illustrating the region-specific nature of exercise-induced thermal responses [8]. Although the present study did not quantify regional muscle activation or mechanical load, the involvement of multiple lower-limb regions during SSGs may contribute to the heterogeneous skin temperature responses observed. These findings support the assessment of specific anatomical regions rather than relying exclusively on a single mean temperature value for the entire lower limb.

In contrast to the sex-related differences observed in skin temperature responses, bilateral thermal asymmetry showed no significant Sex × Time interactions in the anterior thigh, posterior thigh, anterior leg, or posterior leg. Recent evidence in national level cross-country athletes supports this finding. Cabizosu et al. reported no significant sex differences in bilateral thermal asymmetry across the quadriceps, hamstrings, anterior leg, and calf regions, despite observing sex-related differences in absolute Tsk in some thigh regions [27]. Thus, the temporal pattern of bilateral thermal asymmetry was comparable between women and men throughout the assessment period. Healthy athletes have generally been reported to exhibit relatively small temperature differences between homologous contralateral regions [17,28], supporting the potential usefulness of bilateral comparisons when interpreting thermographic assessments.

Moreover, the absence of significant Sex × Time interactions for thermal asymmetry suggests that this variable may provide information that is distinct from the magnitude of exercise-induced skin temperature changes. Previous studies have reported inconsistent findings regarding the relationship between thermal asymmetry and neuromuscular characteristics or physical performance. While some studies have reported no association with isokinetic imbalances, others have identified associations with explosive performance variables such as the rate of force development [29,30]. Therefore, thermal asymmetry should be considered a complementary thermographic variable rather than an isolated indicator of fatigue, neuromuscular imbalance, or injury risk.

From a practical perspective, the present findings suggest that sex and assessment timing should be considered when interpreting post-exercise thermographic measurements in soccer players. The different temporal Tsk responses observed between women and men indicate that group-specific response patterns may need to be considered rather than applying the same post-exercise reference values to both groups. These observations should, however, be interpreted within the methodological conditions of the present protocol and should not be considered evidence of an independent biological effect of sex. Standardising the timing of image acquisition is also important when comparing thermographic assessments across players or sessions. IRT may therefore provide complementary information on post-exercise thermal responses, but it should not be used as a stand-alone indicator of training load or recovery status.

The present study has several strengths, including the direct comparison of women and men soccer players using a standardised IRT protocol and a soccer-specific exercise task. Nevertheless, several limitations should be acknowledged. Individual heart-rate recordings were not retained and therefore could not be analysed as an internal-load outcome. Although individual sRPE data were retained, sRPE was not included in the present analysis. External load was not quantified. Therefore, differences in relative exercise load may have contributed to the observed Tsk responses and cannot be separated from the observed between-group differences.

Body composition may also have influenced the between-sex differences, as women showed a higher mean body fat percentage. The reported group-level mean and standard deviation were calculated from the original individual measurements; however, because body fat percentage was used solely for descriptive characterisation, the individual values were subsequently removed from the analytical database and were not retained for inclusion as a covariate. The menstrual cycle phase was not controlled, and core temperature, cutaneous blood flow, sweating responses, and metabolic heat production were not measured. Consequently, these uncontrolled factors may have contributed to the observed Tsk responses, and the differences between women and men should not be interpreted as an independent effect of biological sex or as evidence of specific thermoregulatory mechanisms.

Exercise intensity was based on age-predicted HRmax rather than directly measured maximal or peak heart rate. In addition, the convenience sample and absence of an a priori sample size calculation may have limited the ability to detect smaller effects, particularly for thermal asymmetry outcomes.

Several methodological aspects of the thermographic protocol should also be considered. The thermographic camera model was not specifically designed for human body assessment, although similar devices have been used in previous studies examining exercise-induced changes in skin temperature [7]. The Post assessment was a non-stabilised measurement that began approximately 5 min after completion of the SSG, without an additional 10–15 min thermal stabilisation period. Consequently, clothing, local heat retention, mechanical pressure, physical contact, and friction may have influenced regional Tsk. These potential artefacts cannot be quantified or retrospectively removed from the dataset. Therefore, the Post measurements should be interpreted as non-stabilised skin temperature responses obtained approximately 5 min after exercise under the specific acquisition protocol used in this study rather than as immediate or fully stabilised post-exercise Tsk values.

The relatively high humidity (86%) may have affected heat dissipation, limiting the generalisability of the findings to other environmental conditions. Finally, thermal assessments were restricted to Pre, Post, and Post_10, preventing a detailed characterisation of post-exercise recovery kinetics. Future studies should include additional recovery time points and integrate IRT with physiological and mechanical measures.

## 5. Conclusions

The present study showed different temporal patterns of skin temperature responses between women and men following a soccer-specific SSG. Women exhibited greater increases in Tsk from Pre to Post and greater net changes from Pre to Post_10, whereas no between-sex differences were observed in the change occurring during the Post-to-Post_10 recovery interval. Bilateral thermal asymmetry showed comparable temporal patterns between sexes. These findings indicate that sex and assessment timing should be considered when interpreting post-exercise Tsk responses using IRT. However, given the potential influence of uncontrolled confounding factors and the methodological constraints of the post-exercise thermographic protocol, the observed differences cannot be attributed independently to biological sex. Further studies using standardised post-exercise acquisition procedures and incorporating external-load, body-composition, menstrual-cycle, and direct physiological measures are needed to clarify these findings.

## Figures and Tables

**Table 1:** Participant characteristics (mean ± standard deviation).

Characteristic	Women (*n* = 22)	Men (*n* = 24)	*p* Value
Age (years)	23 ± 4	24 ± 8	0.708
Body mass (kg)	61.0 ± 8.5	72.6 ± 11.5	<0.001
Height (cm)	158.1 ± 5.5	176.7 ± 7.0	<0.001
Body mass index (kg·m^−2^)	24.3 ± 2.7	23.1 ± 2.5	0.129
Body fat (%)	24.5 ± 5.5	18.2 ± 2.5	<0.001

Data are presented as mean ± standard deviation (SD). *p* values refer to comparisons between women and men participants.

**Table 2:** Skin temperature: Sex × Time interactions and between-sex differences in change.

ROI	Sex × Time Wald χ^2^	Holm *p*	Pre→Post Δ difference, °C (95% CI)	Holm *p*	Post→Post_10_ Δ difference, °C (95% CI)	Holm *p*	Pre→Post_10_ Δ difference, °C (95% CI)	Holm *p*
RAT	33.72	<0.001	1.26 (0.66, 1.86)	<0.001	0.44 (−0.15, 1.04)	0.146	1.70 (1.11, 2.30)	<0.001
LAT	29.78	<0.001	1.16 (0.58, 1.74)	<0.001	0.38 (−0.19, 0.96)	0.192	1.54 (0.97, 2.12)	<0.001
RAL	30.19	<0.001	1.40 (0.79, 2.00)	<0.001	0.13 (−0.47, 0.73)	0.670	1.53 (0.92, 2.13)	<0.001
LAL	34.16	<0.001	1.55 (0.92, 2.19)	<0.001	0.15 (−0.48, 0.78)	0.647	1.70 (1.07, 2.33)	<0.001
RPT	18.26	<0.001	0.86 (0.30, 1.41)	0.005	0.30 (−0.25, 0.85)	0.281	1.16 (0.61, 1.71)	<0.001
LPT	26.27	<0.001	1.08 (0.56, 1.60)	<0.001	0.19 (−0.33, 0.71)	0.476	1.27 (0.75, 1.79)	<0.001
RPL	20.40	<0.001	0.92 (0.35, 1.49)	0.003	0.36 (−0.21, 0.93)	0.220	1.28 (0.70, 1.85)	<0.001
LPL	23.80	<0.001	0.86 (0.31, 1.41)	0.004	0.48 (−0.06, 1.03)	0.083	1.35 (0.80, 1.89)	<0.001

Note: ROI = region of interest; RAT = right anterior thigh; LAT = left anterior thigh; RPT = right posterior thigh; LPT = left posterior thigh; RAL = right anterior leg; LAL = left anterior leg; RPL = right posterior leg; LPL = left posterior leg. Values for contrasts represent the estimated difference in change between women and men (women − men), expressed in °C. Positive values indicate a greater increase in women. *p* values were adjusted using the Holm procedure. Post–Pre represents the change from baseline to the non-stabilised Post assessment obtained approximately 5 min after exercise; Post_10–Post represents the change during the 10 min recovery period; Post_10–Pre represents the net change from baseline to Post_10.

## Data Availability

The individual-level skin temperature and anthropometric data supporting this study remain available from the corresponding author upon reasonable request. Individual body fat percentage values are no longer available because body fat percentage was used solely for descriptive characterisation of the sample and the individual values were not retained after calculation of the reported group-level descriptive statistics. Heart rate and sRPE were monitored in real time exclusively to control exercise intensity during the SSG; individual heart-rate recordings were not exported or retained for subsequent analysis.
